# Sociodemographic, behavioral, and environmental factors of child mortality in Eastern Region of Cameroon: results from a social autopsy study

**DOI:** 10.7189/jogh.07.010601

**Published:** 2017-06

**Authors:** Alain K Koffi, Romain S Wounang, Félicitée Nguefack, Seidou Moluh, Paul–Roger Libite, Henry D Kalter

**Affiliations:** 1 Department of International Health, Johns Hopkins School of Hygiene and Public Health, Baltimore, USA; 2 National Institute of Statistics, Yaoundé, Cameroon; 3 University of Yaoundé I, Department of Pediatrics, Yaoundé, Cameroon; 4 Ministry of Health, Yaoundé, Cameroon

## Abstract

**Background:**

While most child deaths are caused by highly preventable and treatable diseases such as pneumonia, diarrhea, and malaria, several sociodemographic, cultural and health system factors work against children surviving from these diseases.

**Methods:**

A retrospective verbal/social autopsy survey was conducted in 2012 to measure the biological causes and social determinants of under–five years old deaths from 2007 to 2010 in Doume, Nguelemendouka, and Abong–Mbang health districts in the Eastern Region of Cameroon. The present study sought to identify important sociodemographic and household characteristics of the 1–59 month old deaths, including the coverage of key preventive indicators of normal child care, and illness recognition and care–seeking for the children along the Pathway to Survival model.

**Findings:**

Of the 635 deceased children with a completed interview, just 26.8% and 11.2% lived in households with an improved source of drinking water and sanitation, respectively. Almost all of the households (96.1%) used firewood for cooking, and 79.2% (n = 187) of the 236 mothers who cooked inside their home usually had their children beside them when they cooked. When 614 of the children became fatally ill, the majority (83.7%) of caregivers sought or tried to seek formal health care, but with a median delay of 2 days from illness onset to the decision to seek formal care. As a result, many (n = 111) children were taken for care only after their illness progressed from mild or moderate to severe. The main barriers to accessing the formal health system were the expenses for transportation, health care and other related costs.

**Conclusions:**

The most common social factors that contributed to the deaths of 1–59–month old children in the study setting included poor living conditions, prevailing customs that led to exposure to indoor smoke, and health–related behaviors such as delaying the decision to seek care. Increasing caregivers’ ability to recognize the danger signs of childhood illnesses and to facilitate timely and appropriate health care–seeking, and improving standards of living such that parents or caregivers can overcome the economic obstacles, are measures that could make a difference in the survival of the ill children in the study area.

The United Nations recently released its 2015 report that found that the global under–five mortality rate has more than halved since 1990 [1]. This encouraging progress may be attributable to at least the two MDGs dedicated to maternal, reproductive, and child health, namely goal four (MDG 4) that aimed to reduce child mortality by two–thirds and goal five (MDG 5) to reduce the maternal mortality ratio by 75% [2].

However, some critics posit that the MDGs failed to address some gaps that need to be tackled in the new universal and transformative post–2015 development agenda. For example, there are persistent disparities in the under–5 mortality rates within countries, which render any country–level MDGs assessment of progress or achievement misleading or less meaningful.

Child survival is described as being more sensitive to the effects of poverty or material deprivation than most other health outcomes [3]. Poor households are more likely to be exposed to diseases, often lack access to safe water and sanitation, cannot afford nutritious diets, and often have no access to good–quality or affordable health care. In Mozambique, Macassa and Burstrom [4] concluded that behavioral and cultural factors also contribute to child mortality. There is extensive literature on the role of poor access to timely and quality health care interventions on child survival [5–7].

In Cameroon, it is estimated that the upward trend in the under–five mortality rate during the 1990s, from 138 deaths per 1000 live births to 150 deaths per 1000, has now reversed, with the rate having decreased to 88 deaths per 1000 in 2015 [1]. Yet, important disparities remain, with the Eastern region of the country experiencing the second highest under–five mortality at 187 deaths per 1000 live births [8].

There has been a recent call from the international community for Cameroon to accelerate the pace of its progress in order to achieve an under–five mortality rate of 25 or fewer deaths per 1000 live births by 2030 [1]. To reach that goal, an understanding of the most important biological causes of child deaths, along with the behavioral and contextual factors that affect child survival, could help Cameroon make appropriate choices for its situation and accelerate the achievement of improved child survival outcomes.

The present manuscript is part of a series drawn from the WHO/UNICEF–supported Child Health Epidemiology Reference Group’s (CHERG) effort to directly measure the causes and determinants of neonatal and child mortality in selected, high–mortality countries such as Cameroon. This paper focuses on the social autopsy (SA) data of the deaths of children 1–59 months of age that occurred from 2007 to 2010 in Doume, Nguelemendouka, and Abong–Mbang districts in the Eastern region of Cameroon.

From a policy perspective, the purpose of this study was to unveil some of the complex and modifiable factors that contribute to child mortality [9] in the study area, known as one of the most impoverished regions of Cameroon [10], that led to its being dubbed “the forgotten region”, for use by health managers to prioritize and design evidence–based child survival interventions.

## METHODS

The fundamental aim of the study was to identify the household, community, and health system factors that contributed to the child deaths that occurred from 2007 to 2010 in Doume, Nguelemendouka, and Abong–Mbang districts of Cameroon.

Information on births and deaths came from the complete birth histories recorded for all interviewed mothers in a baseline census of all a 16 954 households in the three districts undertaken by Population Services International (PSI) from October to December 2010 for a Home–Based Management of Malaria project.

The sampling methodology of the verbal/social autopsy (VASA) study has been fully described elsewhere [11]. In brief, the study universe included 930 deaths of young children (1–59 months of age) from 2007 to 2010 identified by the census birth histories conducted in the last quarter of 2010. The sampling strategy was to minimize the recall period by taking the one most recent under–five years old death in each household with at least one such death in the four years prior to the census, moving back in time over the survey period until the desired sample size of deaths of 660 child deaths was achieved.

The description of the data collection tools and the fieldwork is available in a paper published earlier [11]. The VASA questionnaire chronologically blended the Population Health Metrics Research Consortium (PHMRC) verbal autopsy questionnaire to determine the biomedical cause of death, with the CHERG Pathway Analysis SA questionnaire [12] to inquire about well–child and illness events leading up to a death. The VASA questionnaire was developed in English and, for the study in Cameroon was translated to French, which is understood by the majority of persons in the study area. Only the local terms for key questionnaire items, such as illness signs and symptoms and the names of local traditional and formal health care providers, were phonetically transliterated to six major local languages– Mongo–Ewondo, Maka, Baka, Mpoong moon, Onveng and Abakoum. The translations were then inserted into a CSProX software application (Serpro S.A, Santiago, Chile) that was developed to enable direct, field–based Computer Aided Personal Interview (CAPI) capture of the VASA interview data on a netbook computer.

For the fieldwork, twenty female interviewers who were native speakers of the local languages and had at least a high school education, received 10 days of in–classroom training in the VASA study background, procedures, ethical standards and conduct of the interview on the netbook, followed by 3 days of field practice, all conducted in French and the local languages. The interviewers were split into three groups (one per district) based on their knowledge of the districts and local languages, and their prior involvement during the mortality survey conducted by PSI in 2010. Each team was led by one field supervisor from the National Statistics Institute (NIS) and in addition received two field visits by office supervisors during the forty days of data collection. The interviewers were trained to select as the respondent the person most knowledgeable of the child’s fatal illness and care provided to the child for the illness. The interview covered the fatal illness from onset to death, including for neonatal deaths, the mother’s pregnancy and delivery. Hence, additional eligible respondents were permitted if necessary. In cases with discordant responses among respondents, the main respondent’s answers were considered. Data collection occurred from 5 March to 15 April 2012.

The analysis of data on preventive and curative care followed the same procedures as described in a prior paper [13], and was guided by the following: (a) review of several sociodemographic and household determinants of the deceased children; (b) coverage of key interventions along the continuum of normal child care provided both inside and outside the home; and (c) illness recognition and care–seeking patterns encompassed by the Pathway to Survival model [9,12,14]. All the examined interventions have been shown to be efficacious and effective in promoting child survival and thus are included among the interventions examined by the Lives Saved (LiST) tool [12] or recommended by the World Health Organization (WHO), and so should be accessible to all children. The list and definitions of some operational variables used throughout this paper are provided in Koffi et al. [13].

In addition, a scoring system was developed based on caregivers’ reports of the child’s feeding behavior, activity level and mental status in order to assess the impact of perceived illness severity at illness onset on caregivers’ attempts at care–seeking for their child’s illness. Hence, three independent categories of illness severity were constructed: normal/mild, moderate, and severe. Details of the method were provided in a prior paper [11]. The Cronbach’s alpha coefficients [15] of the summated scores showed values of 0.90 at the onset of the fatal illness and when the decision to seek care was made. This suggested that the items in the scores elicited highly consistent responses, justifying the reliability of the summated scores according to Nunnaly criteria [16].

Separate to that scoring system, we derived a symptom severity scoring system based on the caregivers observed symptoms by using the World Health organizations’ (WHO) Integrated Management of Childhood Illnesses (IMCI) severity grading for the first symptoms as observed. For the illness symptoms that were in the VA instrument but not in the IMCI, two physician authors (HDK, AKK) assigned symptoms as severe (requiring referral to higher level formal care) or possibly severe (requiring formal health care). The listing of the symptoms and their severity scoring are given in **Online Supplementary Document(Online Supplementary Document).**

The VASA study in Cameroon was first approved by the Cameroon National Research Committee, then by the Johns Hopkins School of Public Health’s Institutional Review Board. All respondents provided informed consent before the interview was conducted.

## RESULTS

Interviews were completed for 635 (96.2%) of the 660 child (1–59 months of age) deaths included in the study sample. More than two–thirds of the respondents (74.7%) were mothers, while 10.6% were fathers of the deceased children, 8.7% grand–mothers, and 6% others relatives.

The sociodemographic characteristics of the deceased children are presented in **Table 1**. The median age at illness onset was 12 months (interquartile range IQR:7–24 months), with two–thirds of the deaths occurring either in the post–neonatal (1–11 months of age) period (41.1%) or second–year (26.3%) of life. Median illness duration was 7 days. There were slightly more deaths of females than males, with a male ratio of 96.0. Most (68.8%) of the 635 deceased children were born at home; the majority (58.0%) also died at home.

**Table 1:** Characteristics of 635 deceased children

Characteristics	N*	Percentage
**Median age at illness onset** (in months)	12 (IQR: 7–24)	
**Median illness** **duration** (in days)	7 (IQR: 3–14)	
**Median age at death** (in months)**:**	12 (IQR: 8–24)	
1–11	261	41.1
12–23	167	26.3
24–59	205	32.4
Don’t know	1	0.2
**Sex:**		
Male	311	49.0
Female	324	51.0
**Masculinity ratio**	96	
**Place of birth:**		
Hospital	117	18.4
Other health provider or facility	74	11.7
On route to a health provider or facility	3	0.5
Home	437	68.8
Other	4	0.6
**Place of death:**		
Hospital	137	21.6
Other health provider or facility	45	7.1
En route to a health provider or facility	20	3.2
Home	368	58.0
Other	65	10.2

IQR – interquartile range

*Q1–Q3: First and third quartiles of the interquartile range (IQR).

**Table 2** shows the characteristics of the mother, her domestic partner, and the household. Approximately 80% of the mothers were married or living with a man at the time of the interview; two–thirds (67.7%) entered in union before 20 years of age. About a third of the mothers (29.3%) lost their index child before reaching 20 years of age. More than two–thirds (71.2%) had some primary level of education (1–6 years of schooling). The average household size was 7.4 persons. The occupation most cited for the breadwinner was farmer/agricultural worker (68.3%). About a quarter or less of the households had modern facilities such as electricity, an improved source of drinking water, and sanitation (flush or improved pit toilet). The vast majority (96.1%) of the households used firewood as fuel for cooking. The median travel time to the caregiver’s usual health care center was 30.0 minutes. The median time families had been living continuously in the same community was about 10 years.

**Table 2:** Characteristics of the mother and her household, 635 child deaths

Maternal characteristics	n	Percentage
**Married or living with a man**	509	80.2
**Median age when first married** (years)**:**	18 (IQR: 15–20)	
12–15	131	25.7
16–19	214	42.0
20–44	152	29.9
Don’t know	12	2.4
**Mother’s median age at time of child death** (in years)**:**	24 (IQR: 19–31)	
13–16	56	8.8
17–19	111	17.5
20–24	171	26.9
25–29	114	18.0
30 or more	172	27.1
Don’t know	11	1.7
**Mean years of schooling:**	5.3 (IQR: 4–6)	
0	26	4.1
1–6	452	71.2
>6	146	23.0
Don’t know	11	1.7
Paternal characteristics:		
**Mean years of schooling** (in years)**:**	6.6 (IQR: 5–8)	
0	7	1.1
1–6	239	37.6
>6	186	29.3
Don’t know	203	32.0
**Household characteristics:**		
Main breadwinner – father	430	67.7
Main breadwinner – mother	50	7.9
Main breadwinner – other	155	24.4
Main breadwinner is farmer/agricultural worker	434	68.3
Median years continuously living in community	10 (IQR: 5–20)	
Household size (mean)	7.4 (IQR: 5–10)	
Household has electricity	137	21.4
Use of piped water–in–house water supply	170	26.8
Use of improved sanitation (improved pit for toilet)	71	11.2
Household uses firewood for cooking	610	96.1
Floor of the house made of cement	93	14.7
Median travel time to nearest health facility (min)	30.0 (IQR: 25–60)	

IQR – interquartile range

*IQR: first and third quartiles of the interquartile range (Q1–Q3)

**Figure 1:**
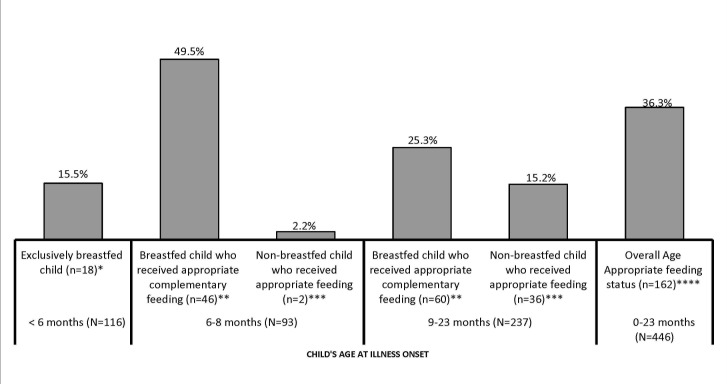
Appropriate feeding for children whose illness started at age 0–23 months. *Child’ illness began before 6 months of age (1-5 months), he/she was being breastfed at the time of fatal illness and was not given anything but breast milk as food. **Breastfed children whose fatal illness started at 6-8 months old and 9-23 months old, and received, respectively, at least two and three complementary non-liquid feedings each day. ***Never-breastfed children whose fatal illness started at 6-8 months old and 9-23 months old, respectively, and received at least four replacement feeds each day (including milk and solid, semi-solid and soft foods). ****Children whose fatal illness started at 0-23 months and satisfied one of the above conditions.

**Figure 1** presents a summary of the nutritional intake before the illness began of the 446 children whose fatal illnesses started between 0–23 months of age. Overall, just 36.3% (n = 162) were being appropriately fed for their age (see **Figure 1**). In more detail, only 15.5% (n = 18) of the 116 children whose fatal illnesses began at 0–5 months of age were exclusively breastfed. Among the 330 children whose illness began at 6–23 months of age, only 32.1% (n = 106) of breastfed children received the recommended complementary non–liquid feeds each day, while 11.5% (n = 38) of non–breastfed children received at least four replacement feeds each day.

**Figure 2:**
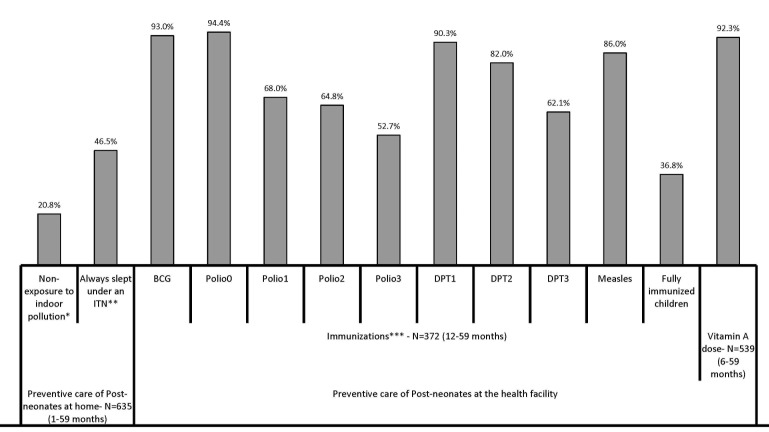
Coverage along the continuum of care for 1-59-month old child deaths in Doume, Nguelemendouka and Abong-Mbang districts, in Eastern Region of Cameroon, from 2007-2010. *Proportion of children who were NOT usually beside or carried by their mother when she cooked inside the home. **Insecticide-treated bed net. ***Information on immunizations was obtained either from the vaccination card or when there was no written record, from the respondent (mainly the mother). Polio0 is the Polio vaccination given at birth; Fully Immunized children received BCG, measles, and three doses each of DPT and polio vaccine (excluding polio vaccine given at birth).

**Figure 2** shows some preventive home care received by the 1–59 months old children along the continuum of care. About one in five children (20.8%) were likely not to be exposed to smoke, ie, he/she was usually away from the mother when she cooked inside the house. Less than half (46.5%) of the children always slept under an insecticide–treated bed net before their fatal illness began.

**Figure 2** further shows the percentage of deceased children 12–59 months of age (n = 372) who received vaccinations against each of the six major preventable childhood diseases by one year of age. Overall, just 36.8% of the children were fully immunized against these diseases before they reached their first birthday. The highest coverage was for Polio 0, BCG, DPT1, and Measles, ranging from 86.0% to 94.4%. The deceased children were least likely to be fully immunized against polio by age one (just 52.7% had had all three doses).

Almost all (92.3%) of the 539 6–59–months–old children received at least one dose of vitamin A before the fatal illness began.

**Figure 3:**
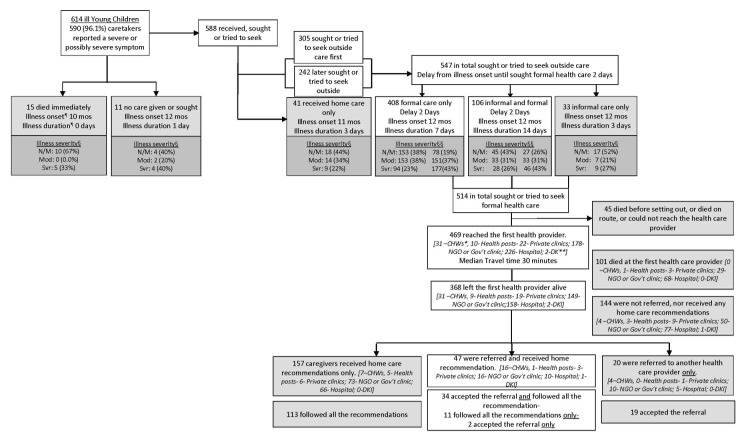
The “Pathway to Survival” for 614 Young Child deaths in Doume, Nguelemendouka and Abong-Mbang districts, in Eastern Region of Cameroon, from 2007-2010. ¶Median values are reported for the age at illness onset, the delay to formal care, and the illness duration due to the skewed values for these variables. §Illness severity at onset. §§Illness severity at onset and when caregiver decided to seek formal care. N/M=normal/mild, Mod=moderate, Svr=severe *CHWs - Trained Community Health worker. **DK Don’t know.

The breakdowns in the Pathway to Survival that contributed to the deaths of the children are presented in **Figure 3**. This analysis included only the 614 children whose caretakers provided information on the types of actions taken for the illness.

Nearly all (96.1%) of the caregivers of the 614 children recognized that their child had a severe or possibly severe sign or symptom when they first noticed that the child was ill. Care was provided or sought for almost all (95.8%, n = 588) of the children; while 15 (2.4%) children were said to have “died immediately,” and no care was given or sought for the other 11 (1.8%).

**Figure 4:**
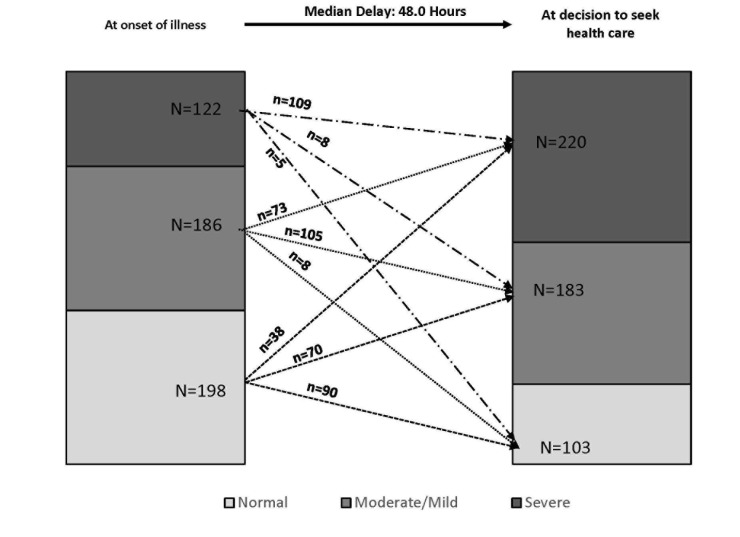
Illness severity ranking at onset and at decision to seek care among children for whom caregivers tried to seek or sought some formal care (N = 506). 8 children had missing information that did not allow their illness severity ranking.

The first action taken for about half (51.9%, n = 305) of the 588 children for whom care was given or sought was to seek care outside the home; the other 283 children (48.1%) first received care inside the home, and 242 of these 283 later sought or tried to seek care outside the home. In total then, 547 (89.1%) of the 614 children for whom care–seeking data was available received, sought, or tried to seek care outside the home. When care was sought outside the home, the majority (74.6%, n = 408) received or tried to seek only formal care, ie, care provide by or at either one of the followings: a trained community health worker (CHW), private doctor or NGO/Government center/post or hospital, 106 (19.4%) received or tried to seek both informal, such as care from a traditional healer or from a pharmacist/drug seller, and formal care, and 33 (6.0%) received informal care only. The median duration (delay) from the illness onset until seeking formal care was 2 days (IQR: 1–3 days) both for those who sought or tried to seek both informal and formal care and those who sought or tried to seek only formal care. For both groups as well, the decision to seek formal care was delayed by 2 days (median time) after the onset of the illness (**Figure 4**), regardless of whether formal care was sought from a health worker in the community or at a hospital or other health facility (χ2(2) = 1.261; *P* = 0.2614). In addition, the median delay was 2 days among children who were perceived to be normal or moderately ill, compared to the median of 2.5 days among those who were severely ill when the decision was made to seek formal care. A median test showed that there was no statistically significant difference in delay between the normal/moderately ill and the severely ill children (χ^2^(1) = 0.831, *P* = 0.3619).

Among those who sought or tried to seek some formal care (N = 514, including 8 who had missing information on perceived illness severity), the percent of children perceived to be severely ill increased from 24.1% (122 out of 506) at the time of illness onset to 43.5% (or 220 out of 506) when the decision was made to seek formal care (**Figure 4**). Many (n = 111) children who were mildly or moderately ill at the time of illness onset, became severely ill by the time their caregivers decided to seek formal care. In other words, the mean illness severity score increased from 1.66 (±SD = 1.043) at onset of the illness to 2.15 (±SD = 0.923) when the decision was made to seek formal care, and the difference of –0.49 (±SD = 0.893) was statistically significant (*t* = –12.35; *P* < 0.0001).

Of the 514 children for whom caregivers tried to seek some formal care, 45 (8.8%) did not reach the health care facility because they died before setting out, died en route or could not reach the health care provider. The remaining 469 (91.2%) children reached the first health care provider after about 30 minutes median travel time, IQR:15–60–minute. Thirty–one (31) went to a community health worker (CHW), 10 to a health post, 22 to a private doctor or clinic, 178 to an NGO or government clinic, and 226 to an NGO or government hospital, and 2 – for which the name or type could not be identified with the available data.

Out of 469 children that reached a first provider, 101 (21.5%) died at that provider. Approximately half (51.5%, or n = 52) of those children who died at the first provider were judged by their caregivers to be severely ill at the time the decision was made to seek formal care. This compares to slightly more than a third (38.6% or 142 out of 367) of those children who left the first provider alive being judged to be severely ill at the time the decision was made to seek formal care. The difference between the two groups (51.5% vs 38.6%) was statistically significant (χ^2^_1_ = 9.325; *P* < 0.010.

In addition, about 39% (n = 144) of the 368 that reached a health care provider and left the provider alive were not referred nor given any home care recommendations. The remaining 224 were either only referred (n = 20) to a second health care provider, only received home care recommendations (n = 157), or were referred and received home care recommendations (n = 47). In summary, just 67 (18.2%) of the 368 that left the first provider alive were referred; however, when recommendations were received, or referrals provided, most of the caregivers (77%–82%) followed all the recommendations or accepted the referral and went to a second health care provider.

**Figure 5:**
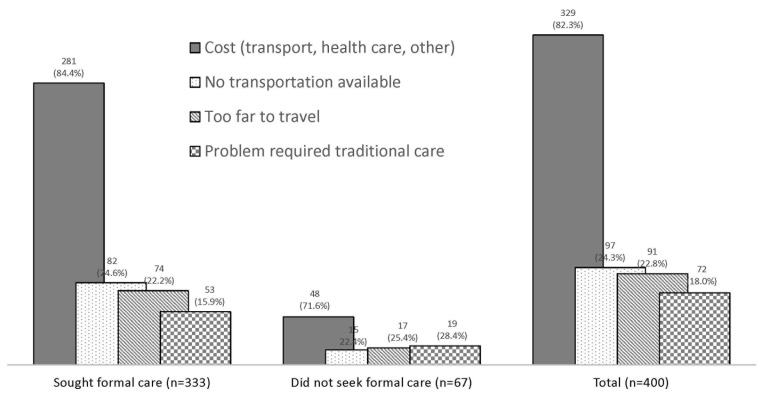
Main care–seeking constraints for child illness (N = 400 caregivers).

**Figure 5** explores the care–seeking constraints for fatal child illnesses. In total, 400 of the 588 caregivers (68.0%) whose children received, sought or tried to seek care reported that they had some concerns or problems in seeking care from a health care provider for their child’s fatal illness. Cost (82.3%), lack of transport (24.3%) and distance (22.8%) were the primary constraints for care–seeking at a health provider, with more caregivers who did not seek care than those who did seek care reporting that they had a concern or problem.

### Discussion

The social autopsy data offer a unique opportunity to assess several households, community and health care system factors related to the children’s deaths. Finding that the majority of children in the study area lived in deprived households concurs with several previous studies that demonstrated that the general health status of children from poor families is compromised by their families’ circumstances [17–19].

The fact that half of the mothers of the deceased children entered into union or marriage at a young age (less than 18 years of age) is of concern. In actuality, child marriage is a violation of human rights [20], because it compromises the development of girls, and often results in early pregnancy and infant mortality. Many countries, including Cameroon, are signatories to all the major child protection conventions, but their application remains uneven. Progress is needed in this area. Besides, improving access to education among girls and eliminating gender gaps in education are known to be important strategies in ending the practice of child marriage [21].

The characteristic of the majority of households in the study area, especially those in rural zones, was typical of the traditional Bantu dwellings made of sun–dried bricks placed in a wooden frame, with Raffia palm fronds or metal roofing. Besides, it was locally and culturally accepted for households to have a single room that serves for cooking during the daytime and as a sleeping room at night. In addition, the overwhelming majority of households relied on domestically available and affordable energy sources, namely firewood for cooking, and kerosene lamps for lighting. Hence, smoke is vented into the home instead of outdoors, leading to 80% of the children being exposed to some of the highest levels of indoor air pollution in the world [22].

According to the World Health Organization, exposure to indoor air pollution more than doubles the risk of pneumonia and other acute lower respiratory infections (ARLI), particularly among children because they may be more vulnerable to the effects of air pollution [23]. Besides, the proportion of child deaths from pneumonia was 15% in Cameroon [24]. The unpublished VASA study report [25] revealed that pneumonia was responsible for 17%–20% of the deaths by expert algorithm and physician–coded analyses among 1–59 months old children in the study setting. This finding sets the stage for more in–depth pollutant exposure research and intervention. Future study considerations should include direct measures to document the amount and composition of pollutant exposures among children. Until then, replacing the traditional 3–rock cook stove with an improved stove and venting the smoke out of the house through a chimney could significantly improve families’ and children's health [26,27].

Deprived or poor households – such as the ones the children were living in before they died—are also known to have increased levels of interrupted breastfeeding and inappropriate complementary/replacement feeding that, in turn, could lead to malnutrition, illness, and mortality [28–30]. The health of about 64% the deceased children whose illness started at 0–23 months old may have also been endangered by their poor nutritional status prior to the illness onset. Indeed, it is estimated that more than one–third of under–five–years–of–age children’s deaths are attributable to undernutrition [31].

When the children became fatally ill, almost all of the caregivers recognized symptoms of severe or possibly severe illnesses. And, unlike the deceased newborn cases [11], a greater number of caregivers (83.7%, n = 514) sought or tried to seek some formal care. The only problem with that was the long delay of 2 days from onset of the illness to when the decision was made to seek formal care. As a result, many (n = 111) children were taken for care only after their illness progressed from mild or moderate to severe.

The delay in deciding to seek care (or so–called delay 1) has been described in previous studies and has been shown to result from an inability to recognize the gravity of the illness condition, or a lack of understanding of disease etiologies and cultural traditions that prescribe seeking treatment first from a traditional healer [32–35]. Some authors have posited that the decision to seek care for childhood illness is largely determined not only by the availability of health care services, but also by social and economic factors, such as religious and cultural norms, the cost of seeking health care, and the acceptability of treatment practices [36]. Other argued that past experience with similar illnesses can motivate mothers to play a 'waiting game' to see whether the illness subsides on its own [37]. The reasons for delay 1 in this study setting are unknown. But we suspect the following conditions hindered a timely decision to seek health care: access to public health care throughout this region is limited, with a median travel time of 30 minutes to the usual health facility for our study population, and rural roads are often of poor quality, especially during the rainy season. In addition, public health facilities in the region often face difficulty maintaining adequate medical personnel and supplies of essential medicines resulting in poor quality of care [38]. Likewise, the current study revealed that unaffordable costs for transportation and health care are key barriers to seeking care in this region, and confirmed findings that suggest a need to mitigate the costs of care–seeking and to provide an effective means of transportation [11].

This delay may have played a major role in the death of the severely ill children who reached the first formal provider and died a few days after. And the fact that this long delay 1 of 2 days did not vary significantly from seeking care at the community or at the facility levels, nor within perceived severity groups at illness onset warrants the need to reinforce the community–based Integrated Management of Childhood Illness (C–IMCI) strategy that improves case management skills of health workers, strengthens the health care system, and addresses family and community practices. The opportunity with C–IMCI to consult a health worker at any time of the day or night, 7 days per week facilitates prompt treatment–seeking and case management [39–41].

The major limitation of this study was the absence of a comparison group that would allow analysis to test whether there were significant differences between the coverage of interventions among cases (deceased children) and controls (alive children). However, the lack of a comparison group in SA studies is common and not so necessary since we are studying interventions that should be accessible to all children [12]. A second limitation refers to the recall period: the median recall period for the 1–59 months deaths was 2 years (IQR: 2–3years). Given that and added to the fact that the respondents were the main caregivers of the deceased newborns, it is possible that the data may have been affected by different types of biases, including recall bias of past events and the likelihood of providing socially desirable answers to sensitive questions. However, the conversational and prompting modes used during the face–to–face interviews, along with the quality of interviewers/supervisor/trainers may have led to better overall recall of events. In addition, the findings in this study in the Eastern region of Cameroon cannot be applied to the whole country. Different regions in Cameroon exhibit marked socio–demographic and cultural variation, disparate levels of economic development and access to health care, as well as distinct climatic conditions and food production likely to affect child health independently of household or neighborhood economic status.

## CONCLUSIONS

In conclusion, as the global health community deliberates the strong likelihood that MDG–4 targets will not be accomplished and about the post–MDG era, it is important that aspects that have been ignored over the past decade find emphasis and support both nationally and globally. The recent social autopsy study conducted in Doume, Nguelemendouka, and Abong–Mbang health districts in the Eastern Region of Cameroon sheds light on the most common household, community and health care system factors that contributed to the deaths of children under five years of age. Among these factors are poor living conditions, poor nutritional status, prevailing customs or cultural practices that lead to exposure to indoor smoke, and health–related behaviors such as delaying the decision to seek care.

Short–term interventions could include the introduction of the C–IMCI program that could increase caregivers’ ability to recognize danger signs of child illnesses and facilitate behavior change for timely and appropriate health care–seeking. Building informed demand among children’s caregivers to seek prompt treatment from an appropriate provider is an important component of any intervention aimed at improving case management coverage. In addition, keeping improved infant and young child feeding high on the public health agenda is crucial to consolidating gains made during the past two decades.

An improved standard of living such that parents or caretakers can overcome the economic obstacles to seeking basic child health care might be efficacious in the long term.
