# Supplementary material for: Sociodemographic, behavioral, and environmental factors of child mortality in Eastern Region of Cameroon: results from a social autopsy study
Source: J Glob Health. 2017 Mar 7;7(1):010601. doi: 10.7189/jogh.07.010601 (PMC5344009; doi:10.7189/jogh.07.010601)
Supplement: Online Supplementary Document [file jogh-07-010601-s001.pdf]

Online Supplementary Document

Koffi et al. Sociodemographic, behavioral, and environmental factors of child mortality in Eastern Region of Cameroon: results from a social autopsy study

J Glob Health 2017;7:010601

SYMPTOM SEVERITY SCORING SYSTEM

	Possibly Severe Illness Sign	Severe Illness Sign
CHILDREN 1-59 MONTHS OF AGE	Fever and Skin rash or Fever alone	Fever and Stiff neck
	Loose stools/ diarrhea for 3 days or more	Loose stools/ diarrhea for 3 days or more
	Visible blood in the loose or liquid stools	Very severe cough
	Cough	Vomit after cough
	Difficult breathing	Indrawing of the chest
	Fast breathing	Stridor
	Skin rash	Grunting
	Blisters containing clear fluid	Wheezing
	Limbs (legs, arms) become very thin	Convulsions
	Swelling in the armpits	Unconscious
		Stiff neck
		Bulging fontanelle
		Swollen legs or feet
		skin flake off in patches
		Hair change in color to a reddish or yellowish color
		Protruding belly
		Lack of blood or pallor
		Whitish rash inside the mouth or on the tongue
		Bleeding from anywhere
		Areas of the skin that turned black
		Road traffic crash/injury
		Fall
		Drowning
		Poisoning
		Bite or sting by a venomous animal
		Burn
		From violence (homicide, abuse)
		Any other injury
